# Molecular Analysis of Carbapenem and Aminoglycoside Resistance Genes in Carbapenem-Resistant *Pseudomonas aeruginosa* Clinical Strains: A Challenge for Tertiary Care Hospitals

**DOI:** 10.3390/antibiotics13020191

**Published:** 2024-02-16

**Authors:** Aamir Jamal Gondal, Nakhshab Choudhry, Ammara Niaz, Nighat Yasmin

**Affiliations:** 1 Department of Biomedical Sciences, King Edward Medical University, Lahore 54000, Pakistan; ajgondal119@gmail.com; 2 Department of Biochemistry, King Edward Medical University, Lahore 54000, Pakistan; nakhshabchoudhry@kemu.edu.pk (N.C.); ammaraniaz@kemu.edu.pk (A.N.)

**Keywords:** *Pseudomonas aeruginosa*, carbapenem resistance, carbapenemases, aminoglycoside-modifying enzyme genes, 16S rRNA methylase variants, sequence types, silver nanoparticles, Pakistan

## Abstract

Carbapenem-resistant *Pseudomonas aeruginosa* (*P. aeruginosa*) strains have become a global threat due to their remarkable capability to survive and disseminate successfully by the acquisition of resistance genes. As a result, the treatment strategies have been severely compromised. Due to the insufficient available data regarding *P. aeruginosa* resistance from Pakistan, we aimed to investigate the resistance mechanisms of 249 *P. aeruginosa* strains by antimicrobial susceptibility testing, polymerase chain reaction for the detection of carbapenemases, aminoglycoside resistance genes, extended-spectrum beta-lactamases (ESBLs), sequence typing and plasmid typing. Furthermore, we tested silver nanoparticles (AgNPs) to evaluate their in vitro sensitivity against antimicrobial-resistant *P. aeruginosa* strains. We observed higher resistance against antimicrobials in the general surgery ward, general medicine ward and wound samples. Phenotypic carbapenemase-producer strains comprised 80.7% (201/249) with 89.0% (179/201) demonstrating genes encoding carbapenemases: *bla*_NDM-1_ (32.96%), *bla*_OXA48_ (37.43%), *bla*_IMP_ (7.26%), *bla*_VIM_ (5.03%), *bla*_KPC-2_ (1.12%), *bla*_NDM-1_/*bla*_OXA48_ (13.97%), *bla*_OXA-48_/*bla*_VIM_ (1.68%) and *bla*_VIM_/*bla*_IMP_ (0.56%). Aminoglycoside-modifying enzyme genes and 16S rRNA methylase variants were detected in 43.8% (109/249) strains: *aac(6′)-lb* (12.8%), *aac(3)-lla* (12.0%), *rmtB* (21.1%), *rmtC* (11.0%), *armA* (12.8%), *rmtD* (4.6%), *rmtF* (6.4%), *rmtB*/*aac(3)-lla* (8.2%), *rmtB*/*aac(6′)-lla* (7.3%) and *rmtB/armA* (3.6%). In total, 43.0% (77/179) of the strains coharbored carbapenemases and aminoglycoside resistance genes with 83.1% resistant to at least 1 agent in 3 or more classes and 16.9% resistant to every class of antimicrobials tested. Thirteen sequence types (STs) were identified: ST235, ST277, ST234, ST170, ST381, ST175, ST1455, ST1963, ST313, ST207, ST664, ST357 and ST348. Plasmid replicon types IncFI, IncFII, IncA/C, IncL/M, IncN, IncX, IncR and IncFIIK and MOB types F11, F12, H121, P131 and P3 were detected. Meropenem/AgNPs and Amikacin/AgNPs showed enhanced antibacterial activity. We reported the coexistence of carbapenemases and aminoglycoside resistance genes among carbapenem-resistant *P. aeruginosa* with diverse clonal lineages from Pakistan. Furthermore, we highlighted AgNP’s potential role in handling future antimicrobial resistance concerns.

## 1. Introduction

*Pseudomonas aeruginosa* (*P. aeruginosa*) strains exhibit exceptional environmental adaptability due to the larger genome making it competent for massive metabolic flexibility, encoding several virulence factors and extensive efflux pump system. *P. aeruginosa* is known as a prominent bacterium involved in healthcare-associated nosocomial infections [1,2,3]. Therapeutic options are severely compromised due to the exploitation of acquired and intrinsic resistance mechanisms by multidrug-resistant (MDR) *P. aeruginosa* [2]. For instance, MDR *P. aeruginosa* strains appeared more efficiently when different classes of antimicrobials were used sequentially [4]. However, the complexities of the underlying resistance mechanisms of MDR *P. aeruginosa* do not neatly correlate with the presence of resistance determinants nor with antimicrobial treatment regimes in clinical settings [4,5]. *P. aeruginosa* core genome carries several transferrable resistance determinants, especially genes encoding for class B carbapenemases or ESBLs, fluoroquinolones and aminoglycoside-modifying enzymes (AMEs) [6,7,8]. Carbapenem-resistant *P. aeruginosa* (CRPA) strains are considered a major health threat due to a high mortality rate of 20–30% [9,10]. The Antimicrobial Testing Leadership and Surveillance (ATLAS) program recorded approximately 20% CRPA prevalence during 2008 to 2018 [11]. The Chinese Antimicrobial Surveillance Network (CHINET) described 25–30% CRPA prevalence from 2005 to 2018 to 20–24% from 2019 to 2021 [12]. The enduring issue of the emergence of carbapenem resistance among *P. aeruginosa* has been growing exponentially over time with a significant global prevalence [12,13,14,15,16]. Therefore, CRPA was classified as one of the three critical priority pathogens by the World Health Organization that demands urgent antimicrobial interventions due to exhausted reserves of antibiotics [17].

Although non-carbapenemase-producing CRPA strains due to decreased outer membrane permeability, overexpression of efflux pump genes or increased chromosomal cephalosporinase AmpC production have been prevalent, carbapenemases have significantly contributed to the expansion of CRPA [18,19,20]. Carbapenemases identified among *P. aeruginosa* are *bla*_NDM-1_, *bla*_IMP_, *bla*_VIM_, *bla*_KPC-2_, *bla*_KPC-90_ and *bla*_OXA-48_ [21,22,23,24,25,26,27,28,29]. Co-resistance of carbapenemases with other resistance-encoding genes in *P. aeruginosa* is usually linked with high levels of carbapenem resistance [17,30]. Thus, the enzymatic modification of aminoglycosides plays a leading role in promoting resistance by inactivating aminoglycosides [31]. Three different families of plasmid or chromosome-mediated aminoglycoside-modifying enzymes (AMEs) are known with variable action mechanisms including acetyltransferases, phosphotransferases and nucleotidyltransferases [32,33]. Another plasmid-encoded resistance mechanism among *P. aeruginosa* is the production of 16S rRNA methylases (RMTs), such as *armA*, *rmtA*, *rmtB*, *rmtC*, *rmtD*, *rmtE* and *npmA* [34]. The co-emergence of aminoglycoside resistance genes, carbapenemases and ESBLs is of great concern due to their rapid transmission by plasmid-mediated horizontal gene transfer [35,36]. Data from Pakistan which would give a clear picture of antimicrobial resistance genes among CRPA are lacking.

The highly variable genome empowers MDR *P. aeruginosa* to survive and disseminate successfully by clonal expansion in addition to the acquisition of various resistance genes [37]. Therefore, the genetic diversity of *P. aeruginosa* helped the global dispersion of high-risk clones, such as ST235, ST111, ST233, ST244, ST357, ST308, ST175, ST277, ST654 and ST298 [38,39]. Most of the high-risk clones have metallo β-lactamase (MBL) carbapenemase production; however, ST111 and ST235 have multiple carbapenemase genes besides MBL [40]. Predominant high-risk clone ST235 represents a diverse geographic distribution with a carriage of ESBLs [41,42,43], carbapenemases [44,45,46,47,48], AME genes [8,45] and chloramphenicol resistance genes [42,49]. ST111 was reported from various European countries, India, the USA and Canada with resistance determinants *bla*_VIM-2_, *bla*_KPC-2_, *bla*_IMP-1_, *bla*_IMP-13_, *bla*_IMP-18_, *bla*_NDM-1_, *bla*_GIM_, *bla*_OXA-2_, *bla*_OXA-17_ and *aac(6′)-Ib* [37,50,51,52,53,54]. Again, insufficient reports regarding the molecular basis for resistance are available from Pakistan. Therefore, the improved knowledge of the transmissible genetic lineages may be helpful in the selection of treatment strategies.

The evolution of epidemic clones jeopardizes all efforts to manage *P. aeruginosa* infections. One of the possible strategy to manage resistance is to use combination treatment, especially with non-antibiotic drugs that may interact with antibiotics [55,56]. In this context, a prime non-antibiotic treatment is the use of silver nanoparticles (AgNPs) with powerful bactericidal properties against MDR microbes [57]. AgNPs facilitate antimicrobial uptake into bateria and lower the desired antibiotic dose required resulting in enhanced antimicrobial activity [58]. The antimicrobial response of AgNPs against MDR *P. aeruginosa* is evident from several reports [59,60,61]; however, CRPA has not been studied before. 

Given the little information available from Pakistan regarding the contribution of different mechanisms in resistance development among *P. aeruginosa* strains, we aimed to study the local circulation of carbapenemases, AMEs and RMT resistance genes and the molecular mechanisms that are responsible for rapidly evolving CRPA isolates from Pakistan. Furthermore, we investigated the effect of AgNPs on the in vitro antimicrobial activity of carbapenems and aminoglycosides against CRPA clinical isolates.

## 2. Results

### 2.1. Characteristics of Bacterial Strains and Antimicrobial Resistance Profile

**Figure 1:**
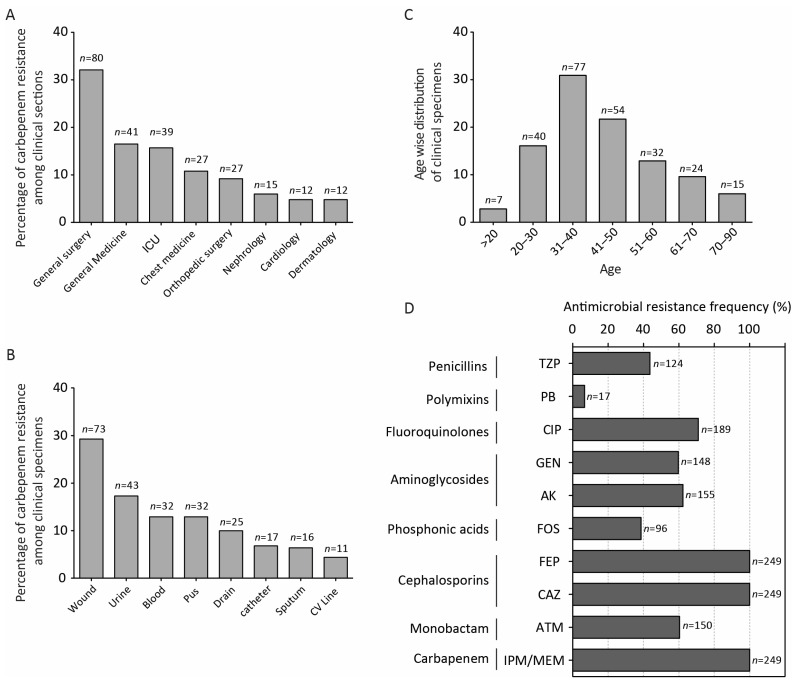
Distribution of clinical strains among (**A**) hospital wards, (**B**) sampling source, (**C**) age groups and (**D**) antimicrobial susceptibility pattern. Abbreviations: ATM, Aztreonam; CAZ, Ceftazidime; FEP, Cefepime; FOS, Fosfomycin; AK, Amikacin; GEN, Gentamycin; CIP, Ciprofloxacin; PB, Polymyxin B; TZP, Piperacillin Tazobactam.

Carbapenem-resistant clinical strains of *P. aeruginosa* (CRPA) were collected from different tertiary healthcare facilities in Lahore from 20 March 2022 to 11 April 2023. Out of 249 strains, 54.2% (*n* = 135) were obtained from female patients while 45.8% (*n* = 114) were from males. The clinical strains were retrieved from different clinical wards, sampling sources and age groups (Figure 1A–C). Antimicrobial susceptibility profile showed higher resistance against commonly used antimicrobials as shown in Figure 1D.

**Figure 2:**
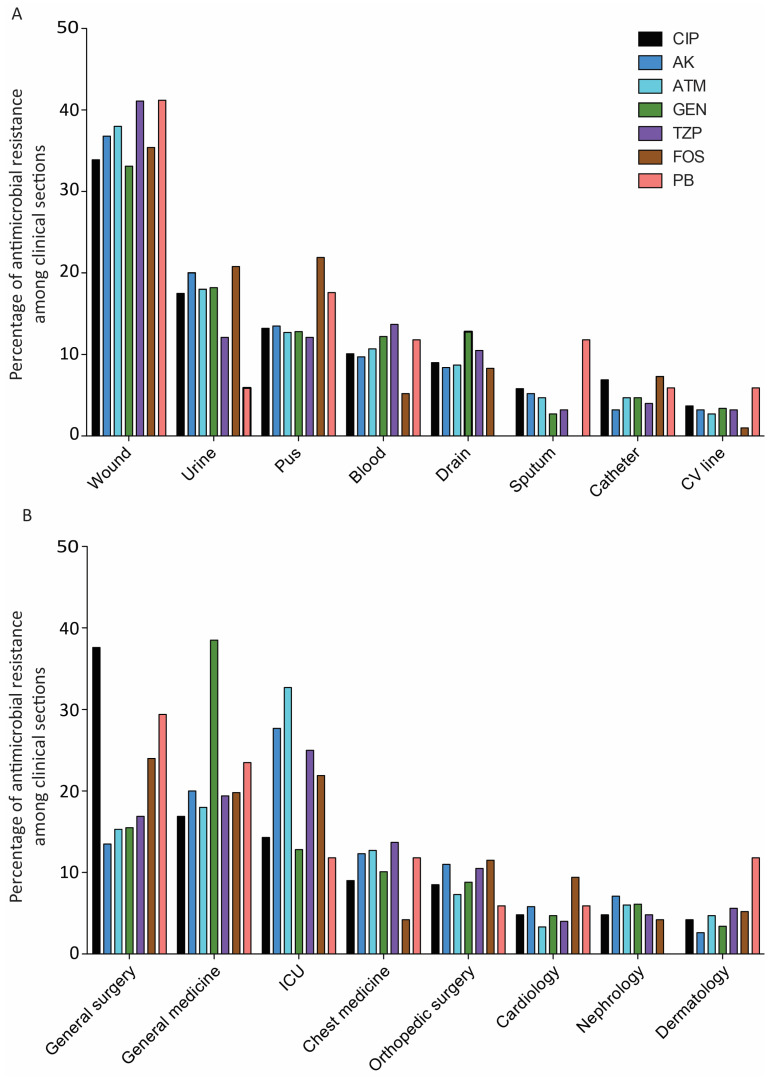
Dissemination of antimicrobial resistance in relation to (**A**) specimen types and (**B**) hospital wards. Abbreviations: ATM, Aztreonam; FOS, Fosfomycin; AK, Amikacin; GEN, Gentamycin; CIP, Ciprofloxacin; PB, Polymyxin B; TZP, Piperacillin Tazobactam.

The pattern of antimicrobial resistance dissemination in relation to hospital wards and specimen type demonstrated that higher resistance against antimicrobials was observed in wound samples while higher resistance of ciprofloxacin (CIP) and gentamycin (GEN) was observed in the general surgery ward and general medicine ward, respectively. The results are shown in Figure 2.

### 2.2. Genomic Analysis of Antimicrobial Resistance Genes

A total of 80.7% (*n* = 201) of CRPA clinical strains were phenotypically carbapenemase-producer strains and 19.3% (*n* = 48) were non-carbapenemase-producer strains. Among carbapenemase-producing strains, 89.0% (*n* = 179) were positive for carbapenemase-encoding genes including *bla*_NDM-1_, *bla*_OXA-48_, *bla*_IMP_, *bla*_VIM_, *bla*_KPC-2_, *bla*_NDM-1_/*bla*_OXA48_, *bla*_OXA-48_/*bla*_VIM_ and *bla*_VIM_/*bla*_IMP_. On the other hand, all strains were identified as ESBL-producer strains. At the molecular level, ESBL-encoding genes *bla*_SHV_, *bla*_CTX-M_, *bla*_TEM_, *bla*_SHV_/*bla*_CTX-M_, *bla*_CTX-M_/*bla*_TEM_, *bla*_SHV_/*bla*_TEM_ and *bla*_SHV_/*bla*_CTX-M_/*bla*_TEM_ were detected, while AME and RMT genes were detected in 43.8% (*n* = 109) strains. The aminoglycoside resistance genes were *aac(6′)-lb*, *aac(3)-lla*, *rmtB*, *rmtC*, *armA*, *rmtD* and *rmtF.* The detailed results of resistance genes are given in Table 1.

The coexistence of carbapenemases and aminoglycoside resistance genes was observed among 43.0% (77/179) of the clinical isolates. The detailed results are given in Table 2.

### 2.3. Genetic Variability Profiling and Replicon Typing Analysis

Genetic diversification of *P. aeruginosa* was determined in terms of clonal lineage analysis and plasmid typing. A total of 13 different sequence types (STs) were identified among the *P. aeruginosa* clinical strains coharboring carbapenem and aminoglycoside resistance genes (*n* = 77). The detected STs were ST235 (32.5%, *n* = 25), ST277 (15.6%, *n* = 12), ST234 (10.4%, *n* = 8), ST170 (10.4%, *n* = 8), ST381 (6.5%, *n* = 5), ST175 (5.2%, *n* = 4), ST1455 (5.2%, *n* = 4), ST1963 (5.2%, *n* = 4), ST313 (3.9%, *n* = 3), ST207 (1.3%, *n* = 1), ST664 (1.3%, *n* = 1), ST357 (1.3%, *n* = 1) and ST348 (1.3%, *n* = 1). It was observed that among the *P. aeruginosa* clinical strains coharboring carbapenem and aminoglycoside resistance genes, 83.1% (64/77) strains were MDR and 16.9% (13/77) were XDR. *P. aeruginosa* clinical strains exhibiting an XDR profile belong to ST235 (*n* = 5), ST170 (*n* = 2), ST313 (*n* = 2), ST234 (*n* = 1), ST277 (*n* = 1), ST348 (*n* = 1) and ST381 (*n* = 1). Furthermore, data from plasmid replicon typing suggested eight different incompatibility groups (Inc) with IncFI, IncFII and IncA/C being the most common among *P. aeruginosa* strains. Other Inc groups included IncL/M, IncN, IncX, IncR and IncFIIK. On the other hand, MOB typing showed that MOBF (F11, F12) was the prevalent plasmid type followed by MOBH (H121) and MOBP (P131, P3). The detailed results of typing are given in Table 2.

### 2.4. Antimicrobial Efficacy of Silver Nanoparticles

*P. aeruginosa* clinical strains coharboring carbapenem and aminoglycoside resistance genes (*n* = 77) were used to check the antimicrobial efficacy of AgNPs in combination with MEM and AK separately. The bacterial cultures were grown in the presence of meropenem (MEM), amikacin (AK), AgNPs, MEM/AgNPs and AK/AgNPs, and MIC values were recorded. Higher MIC values were noted in the bacterial growth when cultured alone in the presence of MEM, AK and AgNPs. However, a reduction in MIC values was observed in the presence of MEM/AgNPs and AK/AgNPs. The results are given in Table 3.

## 3. Discussion

Antimicrobials’ misuse has resulted in resistance development at an alarming rate against commonly used drugs [62,63]. Patients with resistant *P. aeruginosa* infections have poorer prognoses, hence constant monitoring is crucial [64]. Due to the lack of data from Pakistan, we herein collected data on CRPA resistance determinants and analyzed the antimicrobial activity of AgNPs.

The emergence of carbapenem resistance due to carbapenemase production has been considered the prime reason for resistance and treatment failure with adverse economic outcomes [65,66,67,68]. We collected 249 CRPA clinical isolates from March 2022 to April 2023 to analyze carbapenem-resistant mechanisms. Previously, variable rates of CRPA prevalence were observed in Pakistan, such as 81.6% in 2019 [69], 60% in 2014 [70], 57% in 2020 [71], 44% in 2022 [21] and 5.12% in 2023 [72]. This variability might be attributed to lower sample size in such studies. On the other hand, global data showed that CRPA prevalence variations based on different geographic locations such as European and Mediterranean countries (ranging from 10.9% in Sweden to 70.5% in Poland) [20], China (89.4%) [12], Egypt (64.2%) [73], Turkey (20%) [74], India (33%) [75] and Nigeria (40%) [76]. Similarly, a multicenter study in 10 different countries reported 22% [77] and the SENTRY surveillance program 23.9% [78].

CRPA infections are challenging to treat due to the presence of intrinsic and acquired resistance to a number of antimicrobials. We observed that CRPA isolates showed higher resistance against CIP (76.0%), AK (62.3%) and GEN (59.7%). Few reports are available in Pakistan, indicating that carbapenem-resistant strains tend to acquire resistance against aminoglycosides for GEN (74.6%, 30%) and AK (81.8%, 57%, 10%) [70,71,79], while variable aminoglycosides rates among CRPA were observed globally such as AK (93.06%, 83.2%, 44.04% 30.4%) and GEN (85.88%, 87.9%, 59.36%, 35.3%) [14,80,81,82,83]. The presence of aminoglycoside resistance among CRPA indicates an urgent need to identify such strains to avoid therapeutic failures and devise early antibiotic treatment strategies.

Our data revealed that the main reservoirs of CRPA were the general surgery ward, general medicine ward and ICU, while the ICU was reported as the main recovery site of CRPA previously [3,73,80,84]. Urine and wound samples were observed to be the common source of CRPA infection as observed previously [73,85]. However, other sources reported including blood, cerebrospinal fluid, respiratory tract, musculoskeletal and genitourinary infections [3,14,18]. The predominant age group in our study was 31–40 years. Our data are in contrast with previously reported age groups such as 41–60 years from Pakistan [72], >60 years of age from China [12,79], 46–60 years from India [86] and 45–65 years from England [87]. It is evident from these reports that CRPA infections are frequently reported in old age group; however, its incidence among age groups 31–45 years and <20 years has been described in India and Oman [86,88]. 

Non-carbapenemase-producing carbapenem resistance mechanisms have been more prevalent among *P. aeruginosa*; however, the current emergence of carbapenemases is playing a critical role in resistance development [18,20,89,90,91]. It has been reported that carbapenemase-producing CRPA infections are associated with higher mortality rates than non-carbapenemase-producing CRPA infections [18]. Our analysis demonstrated 80.7% carbapenemase producer CRPA isolates, which is higher as compared to global reports such as 25.07% and 33% from 17 health facilities in 12 countries [90,91]. However, variable rates are reported in Pakistan: 18.4% and 52% [21,92]. The variability observed among carbapenemase-producing CRPA prevalence might be due to the deficient testing efforts for detecting *P. aeruginosa* carbapenemase production suggesting a possible higher prevalence than reported [91].

Molecular screening for the carbapenemases in our study identified carbapenemase-encoding genes, *bla*_NDM-1_, *bla*_OXA-48_, *bla*_IMP_, *bla*_VIM_, *bla*_KPC-2_, *bla*_NDM-1_/*bla*_OXA48_, *bla*_OXA-48_/*bla*_VIM_ and *bla*_VIM_/*bla*_IMP_, while aminoglycoside resistance genes among carbapenemase-producing CRPA were *aac(6′)-lb*, *aac(3)-lla*, *rmtB*, *rmtC*, *armA*, *rmtD* and *rmtF*. Previous reports from Pakistan detected *bla_NDM-1_, bla_IMP_, bla_VIM_, bla_OXA-48_* carbapenemases in CRPA [21,71,72,85,93,94]. These findings are supported by various global reports [14,73,90,95,96,97,98,99,100,101]. On the other hand, the detection of *bla*_KPC_ among CRPA was reported infrequently in Pakistan [94]; however, an increasing trend in the global dissemination of *bla*_KPC_-harboring *P. aeruginosa* has been observed [22,28,102,103,104,105,106,107,108]. To the best of our knowledge, the aminoglycoside resistance genes among carbapenemase-producing CRPA were not detected previously from Pakistan; however, one report described the presence of *aph*(3′)-IIb and *aac*(6′)-II among *P. aeruginosa* isolates [109]. It has been reported that AME-encoding gene *aac*(6′)-II is significantly associated with GEN resistance in *P. aeruginosa* [110]. Moreover, several global reports described that the aminoglycoside resistance genes are involved in conferring resistance to *P. aeruginosa* [73,111,112,113,114].

Our data showed the coexistence of carbapenemases and aminoglycoside resistance genes among CRPA clinical isolates. This finding is uncommon, as only a few reports are available that have identified such an association in Korea, Sweden and Greece [115], including *bla*_NDM-1_/*rmtC*, *bla*_NDM-1_/*rmtC/rmtF*, *bla*_NDM-1_*/rmtC/bla*_TEM-1_*/bla*_CTX-M-15_ from India [116], *rmtD*/*bla*_SPM-1_ from Brazil [117,118] and *bla*_NDM-1_/*bla*_VIM_/*rmtB*, *bla*_NDM-1_/*rmtB*/*aac(6′)lb*, *bla*_NDM-1_/*rmtF*/*armA* from Egypt [73]. However, coharbored resistance genes are reported among other species worldwide, such as *armA/bla*_VIM-1_ in *K. pneumoniae* [119], *armA*/*bla*_KPC-2_ in *K. pneumoniae*, [120] and *armA*/*bla*_NDM-1_ in *E. coli*, *K. pneumoniae* and *P. stuartii* [121,122,123,124]. These findings pointed out the difficulty in treating CRPA due to the acquisition of multiple resistance-encoding genes.

The composition of the genetic makeup plays a significant role in the accelerated spread of high-risk clones with distinctive geographical locations. We identified 13 different STs coharboring carbapenem and aminoglycoside resistance genes, including ST235, ST277, ST234, ST170, ST381, ST175, ST1455, ST1963, ST313, ST207, ST664, ST357 and ST348. Previous studies from Pakistan described ST3493, ST3494, ST3472, ST3489, ST3491, ST3492 and ST664 among *P. aeruginosa* isolates [93,109]. However, global data showed a diverse range of STs [40,102,125,126]. In our study, two high-risk clones ST235 and ST277 were identified. ST235 has been described as a critical virulent clone with extraordinary properties to obtain mobile genetic elements, thereby involved in the dissemination of multiple resistance-encoding genes with a successful history of global transmission [127,128,129,130,131,132]. On the other hand, ST277 is identified mainly from Brazil with *bla*_SPM-1_ carbapenemase [133,134,135,136,137], with rare reports from the UK and Japan with *bla*_IMP-1_ carbapenemase detection [37,137,138]. Therefore, the detection of ST277 from Pakistan is alarming.

The genomic plasticity of CRPA is exceptional due to the assorted classes of plasmids. We observed eight different plasmid Inc groups IncFI, IncFII, IncA/C, IncL/M, IncN, IncX, IncR and IncFIIK with MOBF, MOBH and MOBP as more prevalent plasmid types. Another study reported IncH1, IncFIB, IncFI, IncL/M, IncX, IncR, IncA/C, IncL/M, IncW, IncColE, IncFIS and MOBP, MOBF, MOBQ11 among *P. aerugonisa* strains [75]. A high prevalence of IncF and IncH plasmid replicon types was reported in ESBL-positive *P. aeruginosa* isolates [139]. However, the Inc groups identified in different microbial species in the presence of aminoglycoside resistance genes are IncL/M, IncN, IncA/C, IncFII, IncF and IncFI [120,121,123,140,141,142,143,144,145]. Therefore, the management of *P. aeruginosa* infections is becoming problematic due to the highly variable genome, resulting in the resistance development against routinely used antimicrobial drugs. 

In this regard, AgNPs earned attention due to their antimicrobial activity with efficient cell membrane penetration [146]. Antimicrobial-loaded nanoparticles have been extensively used for the inhibition of *P. aeruginosa* infections previously [147]. In vitro studies have proved the significant antimicrobial effects on *P. aeruginosa* isolates with effective growth inhibition [146,148]. Specifically, the antimicrobial and nanoparticle combination proved to enhance antimicrobial efficacy, such as AMP/AgNPs possessing better killing efficiency of ampicillin-resistant *P. aeruginosa* isolates [2,149,150]. We observed a significant reduction in the MIC values of CRPA in the presence of MEM/AgNPs and AK/AgNPs.

## 4. Conclusions

Our study contributed to understanding the antimicrobial resistance pattern existing among *P. aeruginosa* clinical isolates from Pakistan. We described the coexistence of carbapenemases and aminoglycoside resistance genes among CRPA with diverse clonal lineages from Pakistan for the first time. Furthermore, augmented antimicrobial activity of MEM/AgNPs and AK/AgNPs was identified, highlighting AgNPs’ potential role in handling future AMR issues. Therefore, constant monitoring efforts are warranted to develop effective strategies for the control of CRPA and to reduce the incidence of untreatable infections in clinical settings.

## 5. Materials and Methods

### 5.1. Sampling and Identification of Clinical Strains

During the period of 20 March 2022 to 11 April 2023, a total of 249 clinical strains of carbapenem-resistant *P. aeruginosa* were identified and collected from patients who attended different tertiary healthcare facilities in Lahore, Punjab, Pakistan. Strains were phenotypically characterized by analyzing colony morphology and Grams’s staining by culturing on MacConkey agar and cysteine lactose electrolyte-deficient media (Oxoid Ltd., Basingstoke, UK) for urine samples. Strains were biochemically characterized by API-20NE (BioMerieux, Marcy-IEtoile, France).

### 5.2. Antimicrobial Susceptibility Profile Analysis

Standard Kirby–Bauer disc diffusion method was used for antimicrobial susceptibility testing by using Mueller–Hinton agar (MHA) (Oxoid, Ltd., Basingstoke, UK), according to the guidelines of “Performance Standards for Antimicrobial Disc Susceptibility Tests; CLSI Supplement M100, 30th Edition”. Antimicrobial discs from different classes were used as follows: Carbapenems: imipenem (IMP, 10 µg) and meropenem (MEM, 10 µg); Cephalosporins: ceftazidime (CAZ, 30 µg) and cefepime (FEP, 30 µg); Monobactam: aztreonam (ATM, 30 µg); Phosphonic acids: fosfomycin (FOS, 50 µg); Aminoglycosides: amikacin (AK, 10 µg) and gentamycin (GEN, 10 µg); Fluoroquinolone: ciprofloxacin (CIP, 5 µg) (Oxoid, Ltd., Basingstoke, UK). The standard broth microdilution method was used for Polymyxin B as per CLSI recommendation (MIC breakpoints; intermediate ≤ 2, resistant ≥ 4). Quality control strains were *E. coli* ATCC 25922 and *P. aeruginosa* ATCC 27853. The categorization of resistance phenotype was carried out according to criteria described by Magiorakos [151]: XDR, resistant to at least one agent in all but susceptible to two or fewer antimicrobial classes; MDR, resistant to at least one agent in three or more antimicrobial categories; PDR, resistant to all antimicrobial classes.

Modified carbapenem inactivation method (mCIM) was used to determine carbapenemase production by bacterial strains [152]. In brief, 2 to 3 bacterial growth colonies were mixed with 2 mL of tryptone soy broth (TSB media; ThermoFischer Scientific, Waltham, MA, USA). Under sterile conditions, MEM disc (10 µg) was added into the bacterial suspension and incubated at 35 °C ± 2 °C for 4 h. Meantime, mCIM indicator strain suspension (carbapenem-sensitive strain; *E. coli* ATCC 25922) was prepared at a turbidity equivalent to 0.5 McFarland and inoculated on MHA (Oxoid, UK) plate. After 4 h incubation of bacterial strain in TSB media, the MEM disc was transferred to inoculate the MHA plate with indicator strain. Quality control strain *K. pneumoniae* ATCC BAA-1705 was used. The plate was incubated for 18 to 24 h at 35 °C ± 2 °C. CHROMagar^TM^ ESBL media (CHROMagar, Paris, France) was used to identify ESBL-producer strains.

### 5.3. Molecular Identification of P. aeruginosa and Antibiotic Resistance Genes Detection

Genomic DNA was extracted from bacterial cultures by heat lysis method [153]. Briefly, 500 μL sterile dH_2_O was taken in a 1.5 mL microcentrifuge tube and 3–5 bacterial colonies were added to it. Samples were mixed by vortexing for a few seconds. Incubation of bacterial colonies was performed at 98 °C for 10 min at 300 rpm in a thermomixer (Fischerscientific, Waltham, MA, USA). The sample was centrifuged at 1000 rpm for 10 min and the supernatant containing DNA was collected in a new tube. DNA was stored at −80 °C until further processing. The molecular identification of *P. aeruginosa* was performed by polymerase chain reaction (PCR) of 16S rDNA-based primers as described before [154]. Standard PCR was used to detect carbapenem resistance-encoding genes (*bla*_NDM-1_, *bla*_OXA-48_, *bla*_KPC-2_, *bla*_VIM_ and *bla*_IMP_), ESBLs (*bla*_SHV_, *bla*_TEM_ and *bla*_CTX-M_) and aminoglycoside resistance genes (*aac(6′)-lb*, *aac(3)-lla*, *rmtB*, *rmtC*, *armA*, *rmtD*, *rmtF*) [73]. Genomic DNA was amplified in 50 μL reaction volume containing 25 μL of 2x PCR Master Mix (catalog # K0171, Thermoscientific, Waltham, MA, USA), 10 pM of each primer, 300 ng of DNA and dH_2_O up to 50 μL in a thermal cycler (Proflex PCR system, Thermo Fischer Scientific, Waltham, MA, USA). Amplicons were resolved and analyzed by agarose gel electrophoresis (1–1.5%) stained with Syber^TM^ Safe DNA gel stain (catalog # S33102, Thermoscientific, Waltham, MA, USA) and gel documentation system (G:BOX iChemiXT, Syngene, Cambridge, UK). The primer sequences and PCR cycling conditions are given in Table 4.

### 5.4. Determination of bla_NDM_ and bla_KPC_ Alleles

For *bla*_NDM_ and *bla*_KPC_ allele determination, Sanger’s sequencing method was applied by using the BigDye Terminator v3.1 kit for cycle sequencing as per kit recommendations. Cycle sequencing PCR was carried out in 10 μL PCR reaction volume containing 4 μL BigDye terminator 3.1 Ready Reaction Mix, 0.5 μL (3.2 pmol) forward primer, 2 μL purified DNA template (5–20 ng) and 3.5 μL dH_2_O. The following PCR cycling conditions were used: 96 °C 1 min, 96 °C 10 s, 50 °C 5 s, 60 °C 2 min (35 cycles). Purification of PCR product was carried out by using the BigDye XTerminator purification kit. The capillary electrophoresis was carried out by Genetic Analyzer (ABI-3500, Thermo Fischer, Waltham, MA, USA). Sequencing analysis software v6.1 and basic local alignment tools (BLAST, NCBI) were used for data analysis and interpretation. CLC Sequence Viewer 7 version 7.0.2 was used for sequence alignment and mutation analysis.

### 5.5. Multilocus Sequence Typing and Plasmid Typing

Multilocus sequence typing (MLST) analysis was performed on selected *P. aeruginosa* strains coharboring carbapenemase resistance encoding genes and AME/RMT genes (*n* = 77). The following seven housekeeping genes were used for amplification and sequencing [155]: acetyl coenzyme A synthetase (*acsA*), shikimate dehydrogenase (*aroE*), GMP synthase (*guaA*), DNA mismatch repair protein (*mutL*), NADH dehydrogenase I chain C, D (*nuoD*), phosphoenolpyruvate synthase (*ppsA*) and anthralite synthetase component I (*trpE*). The amplification PCR was carried out in a 50 μL reaction mixture comprised of 25 μL of 2x PCR Master Mix (catalog # K0171, Thermoscientific, Waltham, MA, USA), 1 μL of each primer (10 pM), 2 μL (2 ng) of DNA and 22 μL dH_2_O in a thermal cycler (Proflex PCR system, Thermo Fischer Scientific, Waltham, MA, USA). Agarose gel electrophoresis (1–1.5%) was used to analyze the amplified product. The amplified product was purified by using a GeneJET PCR purification kit (catalog # K0701, Thermo Fischer Scientific, Waltham, MA, USA) and further subjected to sequencing analysis as described above. The primer sequences used for PCR amplification, PCR sequencing, amplicon size and annealing temperatures are given in Table 4. MLST database https://pubmlst.org/bigsdb?db=pubmlst_paeruginosa_seqdef was used for assigning sequence types [156] (accessed on 6–22 July 2023).

Plasmid DNA was extracted from a single colony of *P. aeruginosa* by using the plasmid isolation kit (ThermoFischer Scientific, Waltham, MA, USA). Plasmid classification was performed according to their incompatibility groups by using the PCR-based replicon typing (PBRT) method as described previously [157]. Furthermore, Degenerate Primer MOB Typing was used for the classification of γ-proteobacterial transmissible plasmids in five phylogenetic relaxase MOB families (MOB_F_, MOB_P_, MOB_Q_, MOB_H_ and MOB_C_) [158].

### 5.6. Evaluation of Antimicrobial Activity of AgNPs

The broth microdilution checkerboard method was used to evaluate the antimicrobial activity of AgNPs, MEM and AK against the *P. aeruginosa* strains coharboring carbapenem and aminoglycoside resistance genes. AgNPs were purchased from Sigma (Cat # 730785, Sigma-Aldrich, St. Louis, MO, USA). The particle size of AgNPs was 10 nm with a solution concentration of 20 μg/mL in aqueous buffer containing sodium citrate as a stabilizer. MEM, AK and AgNP dilutions were prepared in Mueller Hinton broth, and bacterial cultures were prepared at a concentration of 0.5 McFarland (10^8^ CFU/mL) and further diluted to 1:100 to reach the final concentration of 10^6^ CFU/mL. In a sterile 96-well microtiter plate, each well was inoculated with 100 μL of diluted bacterial suspension and mixed with antibiotic solution. All tests were conducted in duplicate with a growth control without the addition of antibiotics and with sodium citrate addition. The inoculated microtiter plate was incubated at 37 °C for 18 h. After incubation, the fractional inhibitory concentration index (∑FIC) was calculated by dividing the individual MIC of treatments by MIC of the combination drugs. ∑FIC value lower than 0.5 showed synergistic effect, values between 0.5 and 4.0 indifferent and values above 4 antagonistic effect [159].

## Figures and Tables

**Table 1:** Genomic profile of carbapenem-resistant *P. aeruginosa* clinical isolates.

	Genomic Characterization, *n* (%)	
	Carbapenemase-Producing Strains (*n* = 201)	Non-Carbapenemase-Producing Strains (*n* = 48)
Carbapenemases (*n* = 179)		
*bla*_OXA-48_	67 (37.43)	-
*bla*_NDM-1_	59 (32.96)	-
*bla*_IMP_	13 (7.26)	-
*bla*_VIM_	9 (5.03)	-
*bla*_KPC-2_	2 (1.12)	-
*bla*_NDM-1_/*bla*_OXA48_	25 (13.97)	-
*bla*_OXA-48_/*bla*_VIM_	3 (1.68)	-
*bla*_VIM_/*bla*_IMP_	1 (0.56)	-
ESBLs (*n* = 249)		
*bla*_SHV_	63 (31.34)	14 (29.17)
*bla*_CTX-M_	49 (24.38)	17 (35.42)
*bla*_TEM_	27 (13.43)	8 (16.67)
*bla*_SHV_/*bla*_CTX-M_	29 (14.43)	4 (8.33)
*bla*_CTX-M_/*bla*_TEM_	19 (9.45)	3 (6.25)
*bla*_SHV_/*bla*_TEM_	8 (3.98)	1 (2.08)
*bla*_SHV_/*bla*_CTX-M_/*bla*_TEM_	6 (2.99)	1 (2.08)
Aminoglycoside resistance genes (*n* = 109)		
*aac(6′)-lb*	11 (12.79)	3 (13.04)
*aac(3)-lla*	8 (9.30)	5 (21.74)
*rmtB*	17 (19.77)	6 (26.09)
*armA*	13 (15.12)	1 (4.35)
*rmtC*	9 (10.47)	3 (13.04)
*rmtD*	5 (5.81)	-
*rmtF*	4 (4.65)	3 (13.04)
*rmtB*/*aac(3)-lla*	9 (10.47)	-
*rmtB*/*aac(6′)-lla*	7 (8.14)	1 (4.35)
*rmtB/armA*	3 (3.49)	1 (4.35)

**Table 2:** Molecular and genetic characteristics of carbapenem-resistant *P. aeruginosa* clinical isolates (*n* = 77).

Strain ID	Carbapenemases	AME/RMT Genes	ESBLs	Sequence Typing	Plasmid Typing	
					PBRT	MOB
PA-01	*bla*_OXA-48_	*rmtD*	*bla*_SHV_	277	IncFII, IncFI, IncA/C, IncL/M	F12, P131, H121
PA-03	*bla*_NDM-1_	*aac(6′)-lb*	*bla*_CTX-M_	313	IncFII, IncA/C, IncN	F11, F12, H121
PA-04	*bla*_NDM-1_	*armA*	*bla*_CTX-M_	234	IncFII, IncA/C, IncN, IncL/M	F12, P131, H121
PA-08	*bla*_OXA-48_	*rmtB*/*aac(3)-lla*	*bla*_SHV_ */bla*_CTX-M_	235	IncFII, IncA/C, IncL, IncN	F11, F12, H121
PA-09	*bla*_NDM-1_	*armA*	*bla*_CTX-M_	234	IncFII, IncA/C, IncN	F11, F12, H121
PA-10	*bla*_NDM-1_	*rmtB*	*bla*_SHV_/*bla*_TEM_	235	IncFII, IncA/C, IncL/M	F12, P131, H121
PA-11	*bla*_NDM-1_	*aac(6′)-lb*	*bla*_SHV_/*bla*_CTX-M_	357	IncFII, IncA/C, IncN, IncFIIK	F11, F12, H121
PA-14	*bla*_NDM-1_	*aac(6′)-lb*	*bla*_SHV_/*bla*_CTX-M_	235	IncFII, IncA/C, IncN	F11, F12, H121
PA-15	*bla*_NDM-1_/*bla*_OXA-48_	*rmtB*	*bla*_CTX-M_/*bla*_TEM_	235	IncFII, IncA/C, IncL/M, IncN	F11, F12, H121
PA-23	*bla*_OXA-48_	*rmtB*/*aac(3)-lla*	*bla*_SHV_/*bla*_CTX-M_	235	IncFII, IncA/C, IncL/M	F12, P131, H121
PA-25	*bla*_OXA-48_	*rmtB*	*bla*_SHV_	277	IncFII, IncA/C, IncL/M	F12, P131, H121
PA-26	*bla*_NDM-1_	*rmtB*/*aac(3)-lla*	*bla*_SHV_/*bla*_CTX-M_	313	IncFII, IncA/C, IncN	F11, H121
PA-32	*bla*_NDM-1_/*bla*_OXA-48_	*armA*	*bla*_SHV_	313	IncFII, IncA/C, IncL/M, IncN	F11, P131, H121
PA-33	*bla*_NDM-1_	*rmtB*	*bla*_CTX-M_	277	IncFII, IncA/C, IncN	F11, H121
PA-34	*bla*_OXA-48_	*rmtB*	*bla*_TEM_	235	IncFII, IncA/C, IncL/M	F12, P131, H121
PA-35	*bla*_NDM-1_	*rmtD*	*bla*_SHV_	235	IncFII, IncA/C, IncN, IncL/M	F12, P131, H121
PA-36	*bla*_VIM_	*rmtB*	*bla*_CTX-M_	235	IncFII, IncA/C, IncN	F11, F12, H121
PA-39	*bla*_NDM-1_	*rmtD*	*bla*_SHV_	170	IncFII, IncA/C, IncN	F11, F12, H121
PA-44	*bla*_OXA-48_	*rmtB*/*armA*	*bla*_TEM_	170	IncFII, IncA/C, IncL/M	F12, P131, H121
PA-45	*bla*_NDM-1_	*rmtB*	*bla*_SHV_	235	IncFII, IncA/C, IncFIIK	F12, H121
PA-49	*bla*_OXA-48_	*aac(6′)-lb*	*bla*_SHV_	235	IncFI, IncA/C, IncL/M	F12, P131, H121
PA-51	*bla*_OXA-48_	*rmtF*	*bla*_SHV_	1455	IncFII, IncA/C, IncN	F11, F12, H121
PA-59	*bla*_OXA-48_	*aac(6′)-lb*	*bla*_CTX-M_	170	IncFII, IncA/C, IncL/M	F12, H121, P131
PA-62	*bla*_IMP_	*rmtB*	*bla*_TEM_	235	IncFI, IncA/C, IncN, IncX	F11, H121, P3
PA-64	*bla*_OXA-48_	*rmtB*/*aac(3)-lla*	*bla*_SHV_/*bla*_CTX-M_/*bla*_TEM_	170	IncFI, IncA/C, IncL/M	F12, H121, P131
PA-67	*bla*_VIM_	*rmtB*	*bla*_SHV_	234	IncFII, IncA/C, IncN	F12, H121
PA-68	*bla*_NDM-1_	*armA*	*bla*_CTX-M_/*bla*_TEM_	1455	IncFI, IncA/C, IncN	F11, F12, H121
PA-71	*bla*_KPC-2_	*armA*	*bla*_SHV_	235	IncFI, IncA/C, IncL/M, IncP4	F12, P131
PA-72	*bla*_NDM-1_	*rmtB*	*bla*_CTX-M_	664	IncFII, IncA/C, IncX	F12, H121, P3
PA-73	*bla*_NDM-1_/*bla*_OXA-48_	*rmtB*	*bla*_SHV_	235	IncFII, IncA/C	F12, H121
PA-77	*bla*_OXA-48_	*rmtB*/*aac(3)-lla*	*bla*_SHV_ */bla*_CTX-M_	235	IncFII, IncA/C, IncL/M, IncX	F12, P131, H121, P3
PA-78	*bla*_NDM-1_/*bla*_OXA-48_	*aac(3)-lla*	*bla*_CTX-M_ */bla*_TEM_	170	IncFI, IncA/C, IncN, IncFIIK	F11, F12, H121
PA-80	*bla*_OXA-48_	*rmtB*/*aac(6′)-lla*	*bla*_SHV_ */bla*_CTX-M_	235	IncFI, IncA/C, IncN, IncL/M	F11, F12, P131, H121
PA-81	*bla*_VIM_	*rmtB*/*aac(6′)-lla*	*bla*_SHV_	277	IncFII, IncA/C, IncN,	F11, F12, H121
PA-82	*bla*_OXA-48_	*rmtF*	*bla*_CTX-M_	1963	IncFII, IncA/C, IncL/M, IncN	F11, F12, H121, P131
PA-85	*bla*_NDM-1_	*armA*	*bla*_SHV_	277	IncFI, IncA/C, IncN, IncX	F11, F12, H121, P3
PA-93	*bla*_NDM-1_	*aac(3)-lla*	*bla*_TEM_	1963	IncFII, IncA/C, IncN	F11, F12, H121
PA-95	*bla*_NDM-1_/*bla*_OXA-48_	*armA*	*bla*_CTX-M_	348	IncFII, IncA/C, IncL/M, IncHI1	F12, P131, H121, H11
PA-96	*bla*_NDM-1_/*bla*_OXA-48_	*aac(6′)-lb*	*bla*_SHV_ */bla*_CTX-M_	235	IncFII, IncA/C, IncL/M	F12, P131, H121
PA-99	*bla*_IMP_	*rmtB*	*bla*_TEM_	234	IncFII, IncFIIK, IncA/C, IncN	F11, F12, H121
PA-104	*bla*_NDM-1_	*rmtB*	*bla*_SHV_	1455	IncN, IncFII, IncFIIK, IncA/C	F11, F12, H121
PA-105	*bla*_NDM-1_	*rmtD*	*bla*_SHV_/*bla*_CTX-M_	170	IncN, IncFII, IncA/C	F11, F12, H121
PA-108	*bla*_NDM-1_	*aac(3)-lla*	*bla*_SHV_	277	IncFII, IncN, IncA/C, IncFIIK	F11, F12, H121
PA-113	*bla*_NDM-1_	*aac(3)-lla*	*bla*_SHV_/*bla*_CTX-M_	277	IncFII, IncN, IncA/C	F11, F12, H121
PA-116	*bla*_VIM_	*rmtB*	*bla*_CTX-M_	277	IncN, IncFII, IncA/C	F12, F11, H121
PA-120	*bla*_NDM-1_	*rmtB*	*bla*_SHV_	381	IncFII, IncA/C, IncX	F12, H121, P3
PA-122	*bla*_OXA-48_	*rmtB*/*aac(3)-lla*	*bla*_SHV_	1963	IncFII, IncA/C, IncL/M	F12, P131, H121
PA-127	*bla*_VIM_	*armA*	*bla*_SHV_	170	IncFII, IncA/C	F12, H121
PA-131	*bla*_NDM-1_/*bla*_OXA-48_	*rmtB*	*bla*_SHV_/*bla*_CTX-M_	170	IncFII, IncA/C, IncL/M, IncN	F11, F12, P131, H121
PA-138	*bla*_IMP_	*rmtB*/*aac(3)-lla*	*bla*_SHV_/*bla*_CTX-M_	235	IncFII, IncA/C, IncL/M	F12, P131, H121
PA-139	*bla*_NDM-1_	*armA*	*bla*_SHV_	235	IncFII, IncA/C, IncFIIK	F12, H121
PA-140	*bla*_NDM-1_	*armA*	*bla*_TEM_	235	IncFII, IncA/C	F12, H121
PA-141	*bla*_OXA-48_	*rmtB*/*aac(6′)-lla*	*bla*_CTX-M_/*bla*_TEM_	1455	IncFII, IncA/C	F12, H121
PA-152	*bla*_NDM-1_	*rmtB*/*aac(3)-lla*	*bla*_CTX-M_	235	IncN, IncFII, IncA/C, IncL/M	F11, F12, P131, H121
PA-158	*bla*_NDM-1_	*rmtB*/*aac(6′)-lla*	*bla*_SHV_/*bla*_CTX-M_	235	IncN, IncFII, IncA/C, IncX	F11, F12, H121, P3
PA-162	*bla*_NDM-1_/*bla*_OXA-48_	*aac(6′)-lb*	*bla*_SHV_/*bla*_TEM_	175	IncFII, IncA/C, IncL/M, IncX	F12, P131, H121, P3
PA-163	*bla*_VIM_	*aac(6′)-lb*	*bla*_TEM_	235	IncFII, IncA/C	F12, H121
PA-164	*bla*_OXA-48_	*aac(3)-lla*	*bla*_SHV_	175	IncFII, IncA/C, IncL/M	F12, P131, H121
PA-165	*bla*_OXA-48_	*aac(6′)-lb*	*bla*_SHV_/*bla*_TEM_	175	IncFII, IncA/C, IncN	F11, F12, H121
PA-171	*bla*_OXA-48_	*aac(6′)-lb*	*bla*_SHV_	234	IncFII, IncA/C, IncL/M	F12, P131, H121
PA-175	*bla*_NDM-1_	*rmtB*/*aac(6′)-lla*	*bla*_SHV_/*bla*_CTX-M_/*bla*_TEM_	234	IncFII, IncA/C, IncN	F11, F12, H121
PA-178	*bla*_VIM_	*aac(3)-lla*	*bla*_SHV_/*bla*_CTX-M_	234	IncFII, IncA/C, IncFIIK	F12, H121
PA-183	*bla*_NDM-1_	*rmtB*/*armA*	*bla*_SHV_/*bla*_CTX-M_/*bla*_TEM_	277	IncFII, IncFIIK, IncA/C	F12, H121
PA-184	*bla*_NDM-1_	*rmtC*	*bla*_SHV_/*bla*_CTX-M_	277	IncFII, IncA/C, IncN	F11, F12, H121
PA-185	*bla*_VIM_	*rmtC*	*bla*_SHV_/*bla*_TEM_	277	IncFII, IncA/C, IncN, IncR	F12, H121
PA-189	*bla*_NDM-1_	*rmtD*	*bla*_SHV_/*bla*_CTX-M_	235	IncFII, IncA/C, IncN	F11, F12, H121
PA-194	*bla*_IMP_	*rmtC*	*bla*_SHV_/*bla*_TEM_	381	IncFII, IncA/C, IncL/M	F12, P131, H121
PA-198	*bla*_VIM_	*rmtB*/*aac(6′)-lla*	*bla*_SHV_/*bla*_CTX-M_/*bla*_TEM_	277	IncFII, IncA/C, IncFIIK, IncX	F12, H121, P3
PA-202	*bla*_NDM-1_	*rmtC*	*bla*_SHV_/*bla*_CTX-M_	235	IncFII, IncA/C, IncN, IncR	F12, H121
PA-209	*bla*_NDM-1_/*bla*_OXA-48_	*rmtB*/*aac(6′)-lla*	*bla*_SHV_/*bla*_TEM_	235	IncFII, IncA/C, IncL/M, IncN	F11, P131, H121
PA-211	*bla*_NDM-1_	*rmtB*/*armA*	*bla*_SHV_/*bla*_CTX-M_	235	IncFII, IncA/C, IncN, IncFIIK	F11, F12, H121
PA-218	*bla*_OXA-48_	*rmtC*	*bla*_SHV_/*bla*_CTX-M_	234	IncFII, IncA/C, IncL/M	F12, P131, H121
PA-227	*bla*_OXA-48_	*rmtB*	*bla*_CTX-M_	175	IncFII, IncA/C, IncL/M	F12, P131, H121
PA-231	*bla*_OXA-48_	*rmtB*/*aac(3)-lla*	*bla*_SHV_/*bla*_TEM_	381	IncFI, IncA/C, IncL/M, IncX	F12, P131, H121
PA-243	*bla*_NDM-1_/*bla*_OXA-48_	*aac(3)-lla*	*bla*_SHV_/*bla*_CTX-M_	381	IncFII, IncA/C, IncL/M, IncN	F11, F12, P131, H121
PA-244	*bla*_NDM-1_/*bla*_OXA-48_	*rmtB*	*bla*_SHV_/*bla*_CTX-M_	381	IncFII, IncA/C, IncL/M, IncN	F11, F12, P131, H121
PA-249	*bla*_IMP_	*aac(6′)-lb*	*bla*_CTX-M_	207	IncFII, IncA/C, IncN	F11, F12, H121

**Table 3:** Antimicrobial efficacy of AgNPs/MEM and AgNPs/AK by ∑FIC values. Synergism ∑FIC value lower than 0.5, Antagonism ∑FIC values above 4.0, Indifferent ∑FIC values 0.5–4.0.

	For MEM	For AK
∑FIC Interpretation	Strains ID	Strains ID
Synergism	PA-01, PA-04, PA-09, PA-10, PA-14, PA-15, PA-23, PA-25, PA-26, PA-32, PA-33, PA-34, PA-39, PA-44, PA-45, PA-51, PA-59, PA-62, PA-64, PA-71, PA-72, PA-77, PA-78, PA-81, PA-82, PA-93, PA-95, PA-96, PA-104, PA-105, PA-108, PA-116, PA-120, PA-122, PA-131, PA-139, PA-138, PA-140, PA-141, PA-152, PA-158, PA-163, PA-165, PA-171, PA-175, PA-178, PA-183, PA-184, PA-185, PA-189, PA-202, PA-209, PA-211, PA-218, PA-227, PA-231, PA-243, PA-244, PA-249	PA-01, PA-04, PA-09, PA-10, PA-14, PA-15, PA-23, PA-25, PA-26, PA-32, PA-34, PA-33, PA-35, PA-39, PA-45, PA-51, PA-59, PA-62, PA-64, PA-67, PA-71, PA-72, PA-73, PA-77, PA-78, PA-81, PA-82, PA-93, PA-95, PA-96, PA-99, PA-104, PA-105, PA-108, PA-113, PA-116, PA-120, PA-122, PA-131, PA-138, PA-139, PA-140, PA-141, PA-152, PA-158, PA-163, PA-165, PA-171, PA-175, PA-178, PA-183, PA-184, PA-185, PA-189, PA-194PA-198, PA-202, PA-209, PA-211, PA-218, PA-243, PA-244, PA-227, PA-231, PA-249
Indifferent	PA-03, PA-11, PA-35, PA-49, PA-68, PA-73, PA-99, PA-162, PA-164	PA-03, PA-11, PA-49, PA-68, PA-162, PA-164
Antagonism	PA-36, PA-67, PA-85, PA-113, PA-127, PA-194, PA-198	PA-36, PA-44, PA-85, PA-127

**Table 4:** Primers used for PCR and sequencing.

	Primer Sequences (5′–3′)	Tm (°C)	Product Size (bp)
Molecular Identification of *P. aeruginosa* [154]			
PA-SS-F	F: ggg gga tct tcg gac ctc a R: tcc tta gag tgc cca ccc g	56	956
Carbapenemase Resistance Genes [153]			
*bla*_KPC-2_	F: gct aca cct agc tcc acc ttc R: aca gtg gtt ggt aat cca tgc	55	989
*bla*_NDM-1_	F: ggg cag tcg ctt cca acg gt R: gta gtg ctc agt gtc ggc at	53	476
*bla*_VIM_	F: gat ggt gtt tgg tcg cat a R: cga atg cgc agc acc ag	52	390
*bla*_OXA-48_	F: gcg tgg tta agg atg aac ac R: cat caa gtt caa ccc aac cg	52	438
*bla*_IMP_	F: gga ata gag tgg ctt aay tct c R: ggt tta aya aaa caa cca cc	52	232
ESBLs [153]			
*bla*_SHV_	F: ctt tat cgg ccc tca ctc aa R: agg tgc tca tca tgg gaa ag	55	237
*bla*_TEM_	F: cgc cgc ata cac tat tct cag aat ga R: acg ctc acc ggc tcc aga ttt at	55	445
*bla*_CTX-M_	F: atg tgc agy acc agt aar gtk atg gc R: tgg gtr aar tar gts acc aga ayc agc gg	55	593
Aminoglycoside Resistance Genes [73]			
*aac(6′)-lb*	F: ttg cga tgc tct atg agt ggc ta R: ctc gaa tgc ctg gcg tgt tt	59	482
*aac(3)-lla*	F: ggc aat aac gga ggc gct tca aaa R: ttc cag gca tcg gca tct cat acg	60	563
*rmtB*	F: gct ttc tgc ggg cga tgt aa R: atg caa tgc cgc gct cgt at	59	173
*rmtC*	F: gct gcc ctt tgt att gtc R: aga tgt tgg gtt aag tcc c	55	711
*armA*	F: att ctg cct atc cta att gg R: acc tat act tta tcg tcg tc	53	315
*rmtD*	F: cgg cac gcg att ggg aag c R: cgg aaa cga tgc gac gat	58	401
*rmtF*	F: gcg ata cag aaa acc gaa gg R: acc agt cgg cat agt gct tt	60	589
MLST PCR amplification [155]			
*acsA*	F: acc tgg tgt acg cct cgc tga c R: gac ata gat gcc ctg ccc ctt gat	55	842
*aroE*	F: tggggctatgactggaaacc R: taa ccc ggt ttt gtg att cct aca	55	825
*guaA*	F: cgg cct cga cgt gtg gat ga R: gaa cgc ctg gct ggt ctt gtg gta	55	940
*mutL*	F: cca gat cgc cgc cgg tga ggt g R: cag ggt gcc ata gag gaa gtc	55	940
*muoD*	F: acc gcc acc cgt act g R: tct cgc cca tct tga cca	55	1042
*ppsA*	F: ggt cgc tcg gtc aag gta gtg g R: ggg ttc tct tct tcc ggc tcg tag	55	989
*trpE*	F: gcg gcc cag ggt cgt gag R: ccc ggc gct tgt tga tgg tt	55	811
MLST PCR Sequencing [155]			
*acsA*	F: gcc aca cct aca tcg tct at R: gtg gac aac ctc ggc aac ct		390
*aroE*	F: atg tca ccg tgc cgt tca ag R: tga agg cag tcg gtt cct tg		495
*guaA*	F: agg tcg gtt cct cca agg tc R: tca agt cgc acc aca acg tc		372
*mutL*	F: aga aga ccg agt tcg acc at R: atg act tcc tct atg gca cc		441
*muoD*	F: acg gcg aga acg agg act ac R: ttc acc ttc acc gac cgc ca		366
*ppsA*	F: ggt gac gac ggc aag ctg ta R: tcc tgt gcc gaa ggc gat ac		369
*trpE*	F: ttc aac ttc ggc gac ttc ca R: ggt gtc cat gtt gcc gtt cc		441

Abbreviations: *Klebsiella pneumoniae* carbapenemase gene (*bla*_KPC_); New Delhi metallo beta-lactamase (*bla*_NDM_); beta-lactamase oxacillinase 48 gene (*bla*_OXA-48_); metallo-beta-lactamase Verona integron gene (*bla*_VIM_); beta-lactamase imipenemase gene (*bla*_IMP_); beta-lactamase cefotaxime Munich gene (*bla*_CTX-M_); beta-lactamase sulfhydryl reagent variable gene (*bla*_SHV_); beta-lactamase temoneira gene (*bla*_TEM_); aminoglycoside acetyltransferases (*aac(6′)-lb*, *aac(3)-lla*)*;* 16S rRNA methyltransferase (*rmtB*, *armA*, *rmtC*, *rmtD*, *rmtF)*; acetyl coenzyme A synthetase (*acsA*); shikimate dehydrogenase (*aroE*); GMP synthase (*guaA*); DNA mismatch repair protein (*mutL*); NADH dehydrogenase I chain C, D (*nuoD*); phosphoenolpyruvate synthase (*ppsA*); anthralite synthetase component I (*trpE*).

## Data Availability

The data used to support the findings of this study are available from the corresponding author upon request.
